# Impact of BMI and Cardiorespiratory Fitness on Oxidative Stress in Plasma and Circulating Exosomes Following Acute Exercise

**DOI:** 10.3390/biology13080599

**Published:** 2024-08-08

**Authors:** Aaron L. Slusher, Nishant P. Visavadiya, Brandon G. Fico, Brisamar Estébanez, Edmund O. Acevedo, Chun-Jung Huang

**Affiliations:** 1 Department of Pediatrics, Yale School of Medicine, New Haven, CT 06519, USA; aaron.slusher@yale.edu; 2 Exercise Biochemistry Laboratory, Department of Exercise Science and Health Promotion, Florida Atlantic University, FACSM 777 Glades Road, FH11A-126B, Boca Raton, FL 33431, USA; nvisavadiya@fau.edu (N.P.V.); bfico@fau.edu (B.G.F.); 3 Institute of Biomedicine (BIOMED), University of León, 24071 León, Spain; b.estebanez@unileon.es; 4 Department of Kinesiology and Health Sciences, Virginia Commonwealth University, Richmond, VA 23220, USA; eoacevedo@vcu.edu

**Keywords:** aerobic exercise, body mass index, exercise secretome, physical activity

## Abstract

**Simple Summary:**

The regulation of oxidative stress at rest and in response to a single session of exercise is vital for cardiovascular and metabolic health. However, the lack of regular physical activity and exercise and the presence of obesity are known to negatively impact oxidative stress under both conditions. In the present study, the oxidative stress response in circulation and within exosome-like extracellular vesicles (ELVs) released by contracting skeletal muscle during exercise was examined following maximal intensity treadmill running. As a result, we demonstrate that aerobically untrained individuals with obesity exhibit an altered oxidative stress response to maximal treadmill running compared to aerobically untrained and aerobically trained individuals without obesity. Similarly, body mass index, an index of obesity, but not cardiorespiratory fitness, was associated with an adverse oxidative response at rest and immediately following maximal treadmill running. These findings suggest that obesity, more than improved cardiorespiratory fitness, differentially regulates plasma and circulating ELV indices of oxidative stress prior to and immediately following acute maximal treadmill exercise.

**Abstract:**

The impact of cardiorespiratory fitness (VO_2max_) and obesity on indices of oxidative stress in plasma and circulating exosome-like extracellular vesicles (ELVs) were examined following acute exercise. Indices of oxidative stress in plasma and isolated plasma ELVs were examined in aerobically trained (NW-Tr; n = 15) and untrained (NW-UTr; n = 18) normal-weight individuals and aerobically untrained individuals with obesity (Ob-Utr; n = 10) prior to and immediately following acute maximal treadmill running. Following exercise, ELV flotillin-1 expression (*p* = 0.008) and plasma total antioxidant capacity (TAC; *p* = 0.010) increased more in NW-UTr compared to NW-Tr and Ob-UTr participants, whereas plasma protein carbonyls (PC) decreased more in Ob-UTr compared to NW-Tr and NW-UTr groups. ELV glutathione (GSH) concentrations decreased more in NW-Tr compared to NW-UTr and Ob-UTr participants (*p* = 0.009), whereas lipid peroxidase (LPO) concentrations increased more in Ob-UTr compared to NW-Tr and NW-UTr participants (*p* = 0.003). Body mass index (BMI) was associated negatively with plasma TAC and PC (*p* < 0.05) and positively with ELV LPO concentration responses (*p* = 0.009). Finally, plasma-to-total (plasma + ELV) GSH ratios decreased in Ob-UTr compared to NW-Tr and NW-UTr participants (*p* = 0.006), PC ratios increased in NW-Tr and NW-UTr compared to Ob-UTr subjects (*p* = 0.008), and reactive oxygen/nitrogen species ratios increased in NW-UTr and decreased in Ob-UTr participants (*p* < 0.001). BMI, independently of VO_2max_, differentially regulates indices of oxidative stress within plasma and circulating ELVs prior to and immediately following acute maximal treadmill exercise.

## 1. Introduction

Modern Western societies have contributed to a reduction in habitual physical activity and exercise and an increase in sedentary behavior, which is often accompanied by excessive availability and consumption of nutrient-dense foods [1]. This evolutionary mismatch, in combination with excess caloric consumption, has contributed largely to the accumulation of central and ectopic adipose tissue within an increasingly growing population of adults with obesity in the United States and globally [2,3]. More worrisome, the overproduction of tissue-specific (e.g., adipose and vascular) oxidative stress in individuals with obesity, in part, accelerates the pathology of age-related noncommunicable diseases that are among the leading causes of premature death, such as cardiovascular disease (CVD), diabetes, dementia-related disorders, and various types of cancers [4,5,6]. Regular physical activity and exercise have been shown to attenuate damages associated with oxidative stress across the lifespan [7], serving as a potential mechanism that reduces the risk and delays the onset of obesity- and age-related diseases [8,9]. However, the mechanisms that explain how acute and chronic aerobic exercise improves system-wide oxidative stress dysregulation among sedentary individuals with obesity have yet to be elucidated [10,11].

Skeletal muscle is a well-known endocrine organ capable of secreting at least 600 different bioactive molecules in response to activity-induced contraction [12]. These molecules, referred to as “exerkines”, are secreted into circulation and travel to other tissue, including the adipose tissue, liver, heart, and brain, where they have been shown to exert various site-specific, health-mediating adaptive responses to physical activity and exercise [13,14]. Extracellular vesicles, such as exosomes (30–100 nm in size), represent one such mechanism through which skeletal muscle communicates with nearby organs and tissue types to attenuate disease pathology [10,15,16,17,18,19]. In response to acute and chronic endurance and resistance exercise, various molecules are synthesized and packaged within exosomes inside skeletal muscle and then rapidly and transiently secreted into circulation and transported to various other tissues of the body [14,19,20,21]. Likewise, regular aerobic exercise training has been shown to augment the cardioprotective contents within plasma exosomes, independently of changes to exosome concentrations in circulation [22]. These findings suggest that regular physical activity and exercise are effective behavioral strategies to improve the health-mediating capacity of plasma exosomes upon delivery to and release of cardioprotective agents within various tissue and cell types in response to intense aerobic exercise. 

Several studies have also shown that plasma exosomes serve as a delivery mechanism for indices of oxidative stress [23,24], potentially helping to regulate the antioxidant defense within various tissues and organs impacted by acute exercise during obesity. Although recent evidence suggests that plasma exosome release is blunted in obese compared to normal-weight participants following an acute bout of submaximal treadmill running (60% VO_2max_) [25], the impact of obesity and cardiorespiratory fitness on plasma exosomes and their capacity to regulate the oxidative stress response to acute aerobic exercise remains under-investigated. Therefore, the primary purpose of this study was to investigate the impact of obesity and cardiorespiratory fitness on plasma exosomes, indices of oxidative stress measured in plasma and within circulating exosome-like extracellular vesicles (ELVs), and the ratios of plasma-to-total (plasma + ELV) oxidative stress concentrations following acute maximal treadmill running. The relationships between body mass index (BMI) and VO_2max_ with ELVs and oxidative stress in plasma and within circulating ELVs were also examined. It was hypothesized that increased BMI would be associated with reduced markers of ELVs and increased indices of oxidative stress, whereas indices of ELV markers and antioxidant defense would be greater in individuals with elevated VO_2max_. 

## 2. Methods

### 2.1. Participants

Forty-three healthy males between 18 and 35 years old were recruited to participate in the study. Participants were divided into three groups: individuals with normal weight who were aerobically trained (NW-Tr; n = 15), individuals with normal weight who were aerobically untrained (NW-UTr; n = 18), and individuals with obesity who were aerobically untrained (Ob-UTr; n = 10). Participants with a BMI between 18.5 and 24.9 kg/m^2^ were classified as normal weight, and those with a BMI ≥ 30.0 kg/m^2^ were classified as obese. Aerobic training status was determined according to the following criteria: those who participated in 150 or more minutes of moderate-to-vigorous intensity aerobic exercise per week as determined by a 7-day physical activity record [26] and presented with a VO_2max_ ≥ 55.0 mL·kg^−1^·min^−1^ (procedure described below) were considered aerobically trained; those who participate in less than 150 min of moderate intensity (75 min of vigorous intensity) physical activity per week, including aerobic, anaerobic, or resistance exercise and exhibit a VO_2max_ < 50.0 mL·kg^−1^·min^−1^ were considered aerobically untrained [27]. Prior to study, participants provided their informed consent and completed a medical history questionnaire. Participants were excluded from the study if they had a BMI between 25.0 and 29.9; had been previously diagnosed with inflammatory diseases/conditions, such as cardiovascular disease, chronic kidney or liver disease, or diabetes; or were under the current administration of medication known to alter inflammatory and/or metabolic profiles. In addition, participants who were users of tobacco products (cigarettes, cigars, chewing tobacco) and/or consumed an average of ten or more standard alcoholic beverages per week were excluded. The study was approved by the Institutional Review Board at both Virginia Commonwealth University and Florida Atlantic University. 

### 2.2. Exercise Testing Procedures

Participants arrived at the laboratory 6:00 a.m. following an overnight fast for at least 8 h. Participants were also instructed to abstain from alcohol and caffeine intake for at least 24 h and intense physical activity for at least 48 h prior to the laboratory visit. Upon arrival, each participant confirmed their adherence to the previously mentioned instructions and was familiarized with all instruments and procedures. Immediately thereafter, height and weight were assessed using basic medical devices to assess the participant’s BMI by dividing their weight in kilograms by the square of their height in meters. In addition, waist and hip circumferences were obtained by tape measure. Next, participants were instructed to rest quietly for at least 5 min to obtain resting heart rate (HR) and blood pressure (BP) using an HR monitor (Polar T31, Polar Electro, Kempele, Finland) and sphygmomanometer, respectively. 

Participants then underwent a treadmill exercise test to assess maximal oxygen consumption (VO_2max_) administered in gradation according to our laboratory’s previously described protocols [28]. In brief, the test began with a 3-min warm-up at 3 miles per hour and 0% grade. Following the warm-up, speed was increased to elicit 80% ± 5 beats per minute of the participant’s age-predicted maximal HR (APMHR; 220 − age) within the first 2-min stage (stage 1). During the next 2 min (stage 2), HR was allowed to reach a steady state. After the first 4 min, speed remained constant, and the exercise intensity was elevated by increasing grade 2% every 2 min until the participant reached voluntary exhaustion. In addition, HR and rating of perceived exertion (RPE; Borg’s 15-point scale) were recorded during the final 15 s of every exercise stage. VO_2max_ was determined using the ParvoMedics Metabolic Measurement System (ParvoMedics, Sandy, UT, USA), while rates of oxygen consumption (VO_2_) and carbon dioxide production (VCO_2_) were assessed and averaged every 15 s to calculate respiratory exchange ratio (RER: VCO_2_/VO_2_). Criteria for attaining VO_2max_ included a plateau in O_2_ consumption and two of the following secondary criteria: RER ≥ 1.15, HR within 10 bpm of the participant’s APMHR, and an RPE ≥ 19. 

### 2.3. Plasma ELV Isolation

A 10 mL whole blood sample was drawn from each participant’s antecubital vein prior to and immediately upon completion of the maximal exercise test using a 21G butterfly needle into a tube containing K_2_ ethylenediaminetetraacetic acid (K_2_EDTA) (BD Vacutainer, Franklin Lakes, NJ, USA). Whole blood samples were immediately centrifuged at 3000 rpm for 20 min at room temperature, and plasma supernatant was collected and stored at −80 °C until analysis. The Invitrogen™ exosome isolation kit (4484450, Thermo Fisher Scientific, Waltham, MA, USA) was used to isolate plasma ELVs from a fraction of the stored plasma sample. Briefly, frozen plasma samples were defrosted and centrifuged at 2000× *g* for 20 min to obtain a partially clarified supernatant, which was further centrifuged at 10,000× *g* for 20 min at room temperature for clarified plasma. The 500 µL of clarified plasma was mixed well with 250 µL of 1× phosphate-buffered saline (PBS), followed by 150 µL of the plasma exosome precipitation reagent without proteinase K pretreatment. The samples were vortexed and incubated at room temperature for 10 min and then centrifuged at 10,000× *g* for 5 min to obtain a pellet. The pellet with concentrated exosomes was lysed in 250 µL of Pierce™ RIPA buffer (89900, Thermo Fisher Scientific) containing Halt™ protease and phosphatase inhibitor cocktails (78440, Thermo Fisher Scientific). The protein content of concentrated exosomes was quantified using the Pierce™ BCA Protein Assay Kit. (23225, Thermo Fisher Scientific) and then stored at −80 °C until analysis.

### 2.4. Western Blot Analysis

A total of 40 μg of exosome proteins were mixed with Laemmli sample buffer (1610747, Bio-Rad Laboratories, Hercules, CA, USA) containing 2-mercaptoethanol as a reducing agent and resolved according to molecular weight by SDS-PAGE using AnykD™ Criterion™ TGX stain-free™ protein gel (5678125, Bio-Rad Laboratories). After electrophoresis, the gel was transferred onto a Trans-Blot turbo midi 0.2 µm PVDF transfer membrane (1704157, Bio-Rad Laboratories) using a standard protocol (at current: 1.0 A; 25 V constant for 30 min) of the Trans-Blot^®^ turbo™ transfer system (Bio-Rad Laboratories), according to the manufacturer’s instructions. Membranes were blocked with 5% nonfat dry milk prepared in TBST buffer (Tris-buffered saline, 0.1% Tween-20) at room temperature for 1 h. The following primary antibodies were diluted in 5% nonfat dry milk-TBST buffer and then incubated overnight at 4 °C on a shaker: anti-tumor susceptibility gene 101 (TSG101; 1:1000, sc-136111, Santa Cruz Biotechnology, Dallas, TX, USA); CD63 (1:2000, ab68418) and superoxide dismutase 3/EC-SOD (SOD3; 1:2000, ab80946, Abcam Inc., Cambridge, UK); and Alix (1:2000, 2171), Flotillin-1 (Flot-1; 1:1000, 18634), and nuclear factor erythroid 2-related factor 2 (NRF2; 1:2000, 12721, Cell Signaling Technology, Danvers, MA, USA). After overnight incubation, the membranes were washed in TBST (for 10 min thrice) and then incubated in species-specific HRP-conjugated secondary antibodies (Cell Signaling Technology) in TBST for 2 h at room temperature. Membranes were then washed three times with TBST for 10 min each, and immunoreactive protein reaction was carried out using the SuperSignal^TM^ West Pico PLUS Chemiluminescent Substrate (PI34580, Thermo Fisher Scientific) solutions. A ChemiDoc^TM^ XRS+ (Bio-Rad Laboratories) imaging system was used for protein bands visualization, and the density was quantified by ImageJ software (National Institutes of Health, Bethesda, MD, USA) and normalized to total protein by staining membranes with Ponceau S solution (p7170, Millipore Sigma, Burlington, MA, USA).

### 2.5. Biochemical Analysis

Indices of oxidative stress concentrations within whole plasma and isolated ELVs (described above) were analyzed simultaneously for the following redox biomarkers using commercially available kits. Total antioxidant capacity (TAC value-Trolox equivalent; MAK187, Millipore Sigma) and lipid peroxidation (LPO; ab233471, Abcam Inc.) were analyzed according to the manufacturer’s instructions using the Epoch™ microplate spectrophotometer (BioTek Instruments, Winooski, VT, USA). Reduced glutathione (GSH; 700340, Cayman Chemical, Ann Arbor, MI, USA) and protein carbonyls (PC, 701530, Cayman Chemical) fluorometric assays were performed at 380 nm excitation/530 nm emission and 530 nm excitation/590 nm emission, respectively, using a Synergy HTX spectrofluorometer (BioTek Instruments). Total reactive oxygen species and reactive nitrogen species (ROS/RNS) were quantified by the redox-sensitive fluorescent probe 2′,7′-Dichlorofluorescin diacetate (10 µM, DCFH2-DA, D6883, Millipore Sigma). In short, 10 µL of plasma or ELVs were incubated with 90 µL of DCFH2-DA fluorogenic probe in PBS, pH 7.4, for 10 min in the dark at room temperature. The fluorescence level of oxidized DCF was determined at 485 nm excitation/530 nm emission filters by a Synergy HTX spectrofluorometer (BioTek Instruments). The plasma SOD3/EC-SOD (ELH-SOD3, RayBiotech Inc., Peachtree Corners, GA, USA) and NRF2 (EH348RB, Thermo Fisher Scientific) contents were measured by commercially available ELISA kits. Total protein concentration of plasma and exosomes was determined using the Pierce™ BCA Protein Assay Kit (23225, Thermo Fisher Scientific), and the total protein value was used for data normalization.

### 2.6. Statistical Analyses

Data analysis was performed using the Statistical Package for the Social Sciences (SPSS version 28.0), and normality was examined using the Shapiro-Wilk test. In the event of a statistical outlier, data were excluded from analysis. One-way analysis of variance (ANOVA) tests was conducted to compare anthropometric and cardiorespiratory profiles between the NW-Tr, NW-UTr, and Ob-UTr participant groups. In addition, baseline differences across ELV markers and indices of oxidative stress in plasma and ELVs were also examined by one-way ANOVA. A three-group (NW-Tr, NW-UTr, and Ob-UTr) by twotimepoint (pre and immediately post maximal exercise) repeated measures ANOVA was used to examine the effects of acute aerobic exercise on ELV and plasma measures. If Mauchly’s test indicated a violation of the sphericity assumption, the degrees of freedom were corrected by the Greenhouse-Geisser estimates. To facilitate the presentation of ratios comparing plasma-to-total (plasma plus ELV) levels of NRF2 and SOD3 while avoiding dependence on measurement units, we applied the min-max normalization method. This method, expressed as x′ = (x − min(x))/(max(x) − min(x)), transforms the data to fall within a common range of [0.0, 1.0] to give both variables (derived from ELISA and Western blot) an equal weight. Lastly, Pearson’s correlations were utilized to examine the relationships of BMI and cardiorespiratory fitness (relative VO_2max_) with ELV markers and oxidative stress measured from plasma and ELVs in response to maximal exercise (absolute change from pre- to immediately post-maximal exercise). Importantly, correlational analyses were performed while controlling for BMI and relative VO_2max_ as covariates, and the Benjamini-Hochberg method (False Discovery Rate) was applied to correct for any limitations related to performing multiple comparisons. All data are presented as means ± S.D. unless otherwise stated, with statistical significance defined as a *p*-value ≤ 0.05 balanced by effect size.

## 3. Results

### 3.1. Anthropometric Characteristics and Cardiovascular Measures

Baseline anthropometric characteristics and cardiovascular measures for each group are reported in Table 1. Although no differences were observed in age or height, Ob-UTr exhibited significantly greater body weights (*F*_[2, 40]_ = 74.034, *p* < 0.001, η^2^ = 0.79), BMI (*F*_[2, 40]_ = 112.282, *p* < 0.001, η^2^ = 0.85), waist and hip circumferences (*F*_[2, 40]_ = 74.273, *p* < 0.001, η^2^ = 0.79; *F*_[2, 40]_ = 62.126, *p* < 0.001, η^2^ = 0.76, respectively), and waist-to-hip ratios (*F*_[2, 40]_ = 27.196, *p* < 0.001, η^2^ = 0.58) compared to both NW-Tr and NR-UTr groups. In addition, a stepwise increase in resting HR was observed among NW-Tr, NW-UTr, and Ob-UTr groups (*F*_[2, 40]_ = 13.391, *p* < 0.001, η^2^ = 0.40). Ob-UTr also presented with greater SBP compared to both NW-UTr and NW-Tr groups and a greater DBP compared to NW-UTr participants (*F*_[2, 40]_ = 25.391, *p* < 0.001, 0.56; *F*_[2, 40]_ = 4.068, *p* = 0.025, η^2^ = 0.17, respectively). Finally, while NW-Tr and Ob-UTr participants both exhibited greater absolute VO_2max_ compared to NW-UTr participants, a stepwise decrease in relative VO_2max_ was observed among NW-Tr, NW-UTr, and Ob-UTr groups (*F*_[2, 40]_ = 25.873, *p* < 0.001, η^2^ = 0.56; *F*_[2, 40]_ = 115.422, *p* < 0.001, η^2^ = 0.85, respectively). 

### 3.2. ELV Marker Expression

**Figure 1:**
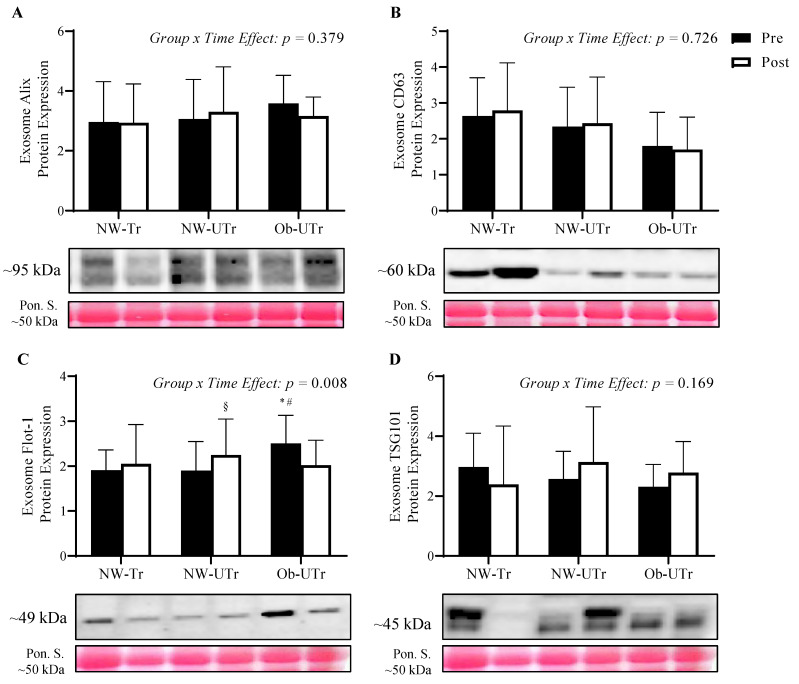
ELV markers prior to and in response to acute maximal treadmill exercise. Repeated measures analysis of variance analysis demonstrated no differences in Alix, CD63, or TSG101 were observed between participant groups (panels **A**, **B**, and **D**). Flot-1 was significantly greater in Ob-Utr compared to NW-Tr and NW-Utr participants prior to exercise, whereas NW-UTr participants exhibited a significantly greater increase in plasma ELV Flot-1 expression from pre-to-post maximal exercise compared to both the NW-Tr and Ob-UTr groups (panel **C**). Data are presented as means ± S.D. and are normalized by Ponceau S solution. The * indicates a significant difference compared to NW-Tr participants; ^#^ indicates a significant difference compared to NW-Utr participants; ^§^ indicates a significant difference compared to pre-exercise values *(p* ≤ 0.05). Abbreviations: ELV: exosome-like extracellular vesicles; Flot-1: Flotillin-1; NW-Tr: individuals with normal weight who are aerobically trained; NW-UTr: individuals with normal weight who are aerobically untrained; Ob-UTr: individuals with obesity who are aerobically untrained; TSG101: tumor susceptibility gene 101. The uncropped western blot figures were presented in Figure S1.

Prior to maximal exercise, baseline ELV Flot-1 expression was significantly greater in Ob-UTr compared to both NW-Tr and NW-UTr participants (*F*_[2, 40]_ = 4.102, *p* = 0.024, η^2^ = 0.17; Figure 1C). No differences were observed among baseline Alix (*F*_[2, 40]_ = 0.818, *p* = 0.449, η^2^ = 0.04; Figure 1A), CD63 (*F*_[2, 40]_ = 1.893, *p* = 0.164, η^2^ = 0.09; Figure 1B), or TSG101 ELV expression levels (*F*_[2, 40]_ = 1.515, *p* = 0.232, η^2^ = 0.07; Figure 1D) in response to maximal exercise. Following maximal exercise, NW-UTr participants exhibited a significantly greater increase in ELV Flot-1 expression from pre-to-post maximal exercise compared to both the NW-Tr and Ob-UTr groups (*Group × Time Effect*: *F*_[2, 40]_ = 5.519, *p* = 0.008, η^2^ = 0.22). No differences among Alix (*Group* × *Time Effect*: *F*_[2, 40]_ = 0.993, *p* = 0.379, η^2^ = 0.05), CD63 (*Group* × *Time Effect*: *F*_[2, 40]_ = 0.322, *p* = 0.726, η^2^ = 0.02), or TSG101 expression levels (*Group* × *Time Effect*: *F*_[2, 40]_ = 1.861, *p* = 0.169, η^2^ = 0.01) were observed following maximal exercise. 

### 3.3. Indices of Plasma Oxidative Stress

**Figure 2:**
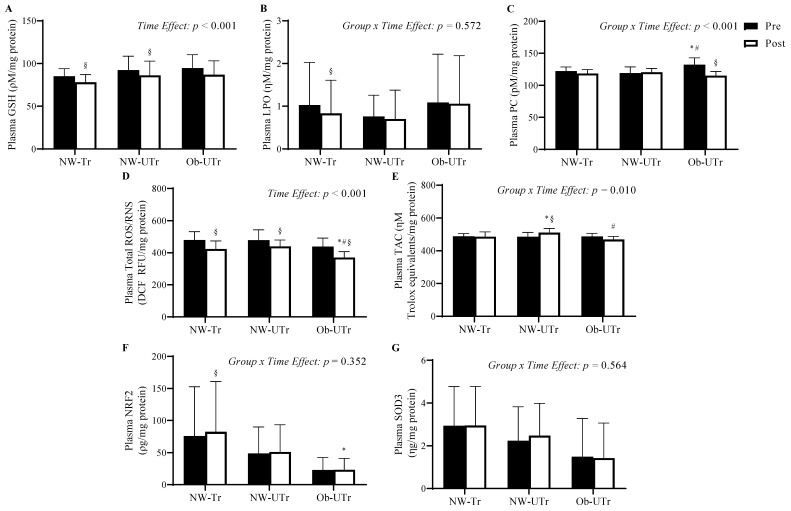
Indices of oxidative stress measured in plasma prior to and in response to acute maximal treadmill exercise. Repeated measures analysis of variance analysis demonstrated a differential impact of acute maximal treadmill exercise on plasma PC concentrations (panel **C**) and TAC values (panel **E**) in NW-Tr, NW-Utr, and Ob-UTr participant groups, whereas plasma GSH (panel **A**) and total ROS/RNS (panel **D**) concentrations responded similarly across all groups. Acute maximal treadmill exercise exhibited no impact on LPO (panel **B**), NRF2 (panel **F**), or SOD3 (panel **G**) concentrations in plasma. Data are presented as means ± S.D. * indicates a significant difference compared to NW-Tr participants; ^#^ indicates a significant difference compared to NW-Utr participants; ^§^ indicates a significant difference compared to pre-exercise values *(p* ≤ 0.05). Abbreviations: GSH: glutathione; LPO: lipid peroxidation; NRF2: nuclear factor erythroid 2-related factor 2; NW-Tr: individuals with normal weight who are aerobically trained; NW-UTr: individuals with normal weight who are aerobically untrained; Ob-UTr: individuals with obesity who are aerobically untrained; PC: protein carbonyl; ROS/RNS: reactive oxygen/nitrogen species; SOD3: superoxide dismutase 3; TAC: total antioxidant capacity.

Baseline plasma PC concentrations were significantly greater in Ob-UTr compared to both NW-Tr and NW-UTr groups (*F*_[2, 39]_ = 7.122, *p* = 0.002, η^2^ = 0.27; Figure 2C). To the contrary, no difference were observed in plasma concentrations for GSH (*F*_[2, 39]_ = 1.626, *p* = 0.210, η^2^ = 0.08; Figure 2A), LPO (*F*_[2, 39]_ = 0.574, *p* = 0.568, η^2^ = 0.03; Figure 2B), total ROS/RNS (*F*_[2, 39]_ = 1.813, *p* = 0.177, η^2^ = 0.09; Figure 2D), TAC(*F*_[2, 39]_ = 0.070, *p* = 0.932, η^2^ = 0.003; Figure 2E), NRF2 (*F*_[2, 39]_ = 2.950, *p* = 0.064, η^2^ = 0.13; Figure 2F), or SOD3 (*F*_[2, 39]_ = 2.109, *p* = 0.135, η^2^ = 0.10; Figure 2G). Following maximal exercise, Ob-UTr participants exhibited a significantly greater reduction in plasma PC concentrations from pre- to post-maximal exercise compared to both the NW-Tr and NW-UTr groups (*Group* × *Time Effect*: *F*_[2, 39]_ = 13.384, *p* < 0.001, η^2^ = 0.41), and NW-UTr participants exhibited a significantly greater increase in TAC values compared to the NW-Tr and Ob-Tr groups (*Group* × *Time Effect*: *F*_[2, 39]_ = 5.235, *p* = 0.010, η^2^ = 0.21). Additionally, plasma GSH concentrations decreased in response to maximal exercise similarly in both the NW-Tr and NW-UTr groups (*Time Effect*: *F*_[2, 39]_ = 26.450, *p* < 0.001, η^2^ = 0.404), whereas total ROS/RNS concentrations decreased similarly across all three groups and were significantly lower in Ob-UTr compared to both the NW-Tr and NW-UTr groups post exercise (*Time Effect*: *F*_[1, 39]_ = 0.37.173, *p* < 0.001, η^2^ = 0.49). No differences in the plasma LPO (*Group* × *Time Effect: F*_[2, 39]_ = 0.566, *p* = 0.572, η^2^ = 0.03), NRF2 (*Group* × *Time Effect*: *F*_[2, 39]_ = 1.073, *p* = 0.352, η^2^ = 0.05), and SOD3 concentrations (*Group* × *Time Effect*: *F*_[2, 39]_ = 0.581, *p* = 0.564, η^2^ = 0.03) were observed among the groups.

### 3.4. Indices of Oxidative Stress from Circulating ELVs

**Figure 3:**
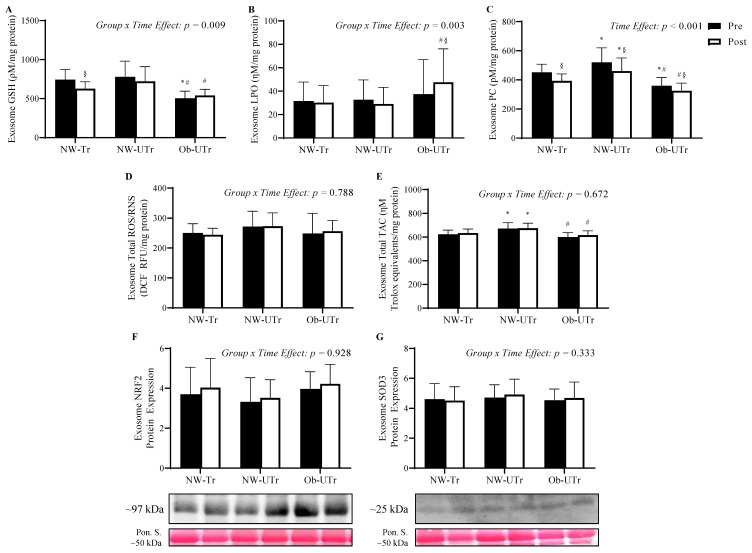
Indices of oxidative stress measured from circulating ELVs prior to and in response to acute maximal treadmill exercise. Repeated measures analysis of variance analysis demonstrated a differential impact of acute maximal treadmill exercise on GSH (panel **A**) and LPO (panel **B**) concentrations measured from circulating ELVs across the NW-Tr, NW-Utr, and Ob-UTr participant groups, whereas PC concentrations from plasma ELV responded similarly across all participant groups (panel **C**). Acute maximal treadmill exercise exhibited no impact on ELV total ROS/RNS (panel **D**), TAC (panel **E**), NRF2 (panel **F**), or SOD3 (panel **G**) expression. Data are presented as means ± S.D. and are normalized by Ponceau S solution (NRF2 and SOD3). The * indicates a significant difference compared to NW-Tr participants; ^#^ indicates a significant difference compared to NW-Utr participants; ^§^ indicates a significant difference compared to pre-exercise values *(p* ≤ 0.05). Abbreviations: ELV: exosome-like extracellular vesicles; GSH: glutathione; LPO: lipid peroxidation; NRF2: nuclear factor erythroid 2-related factor 2; NW-Tr: individuals with normal weight who are aerobically trained; NW-UTr: individuals with normal weight who are aerobically untrained; Ob-UTr: individuals with obesity who are aerobically untrained; PC: protein carbonyl; ROS/RNS: reactive oxygen/nitrogen species; SOD3: superoxide dismutase 3; TAC: total antioxidant capacity. The uncropped western blot figures were presented in Figure S1.

Prior to maximal exercise, baseline GSH concentrations from circulating ELVs were significantly lower in Ob-UTr compared to both NW-Tr and NW-UTr participants (*F*_[2, 40]_ = 10.255, *p* < 0.001, η^2^ = 0.34; Figure 3A). A stepwise increase in PC concentrations was observed in Ob-UTr compared to NW-Tr, and, subsequently, NW-UTr participants (*F*_[2, 40]_ = 13.789, *p* < 0.001, η^2^ = 0.41; Figure 3C). Likewise, TAC values were greater in NW-UTr compared to NW-Tr and Ob-UTr participants (*F*_[2, 40]_ = 11.024, *p* < 0.001, η^2^ = 0.36; Figure 3E). No differences were observed in baseline LPO concentrations (*F*_[2, 40]_ = 0.279, *p* = 0.758, η^2^ = 0.01; Figure 3B), NRF2 expression levels (*F*_[2, 40]_ = 0.999, *p* = 0.377, η^2^ = 0.05; Figure 3F), total ROS/RNS concentrations (*F*_[2, 40]_ = 1.036, *p* = 0.364, η^2^ = 0.05; Figure 3D), or SOD3 expression levels (*F*_[2, 40]_ = 0.135, *p* = 0.874, η^2^ = 0.01; Figure 3G) measured from circulating ELVs prior to maximal exercise.

Following maximal exercise, NW-Tr participants exhibited a significantly greater reduction in circulating ELV GSH concentrations from pre-to-post maximal exercise compared to both the NW-UTr and Ob-UTr groups (*Group* × *Time Effect*: *F*_[2, 40]_ = 5.358, *p* = 0.009, η^2^ = 0.21). To the contrary, Ob-UTr participants exhibited a significantly greater increase in circulating ELV LPO concentrations from pre-to-post maximal exercise compared to both the NW-Tr and NW-UTr groups (*Group* × *Time Effect*: *F*_[2, 40]_ = 6.721, *p* = 0.003, η^2^ = 0.25). Additionally, circulating ELV PC concentrations decreased similarly across all three groups (*Time Effect*: *F*_[1, 40]_ = 35.998, *p* < 0.001, η^2^ = 0.47). No differences in the NRF2 expression (*Group* × *Time Effect*: *F*_[2, 40]_ = 0.075, *p* = 0.928, η^2^ = 0.004), total ROS/RNS concentrations (*Group* × *Time Effect*: *F*_[2, 40]_ = 0.240, *p* = 0.788, η^2^ = 0.01), SOD3 expression (*Group × Time Effect*: *F*_[2, 40]_ = 1.130, *p* = 0.333, η^2^ = 0.05), and TAC values (*Group* × *Time Effect*: *F*_[2, 40]_ = 0.402, *p* = 0.672, η^2^ = 0.02) were observed among the participant groups following maximal exercise. 

### 3.5. Proportion of Plasma Relative to Total Circulating Oxidative Stress Concentrations

**Figure 4:**
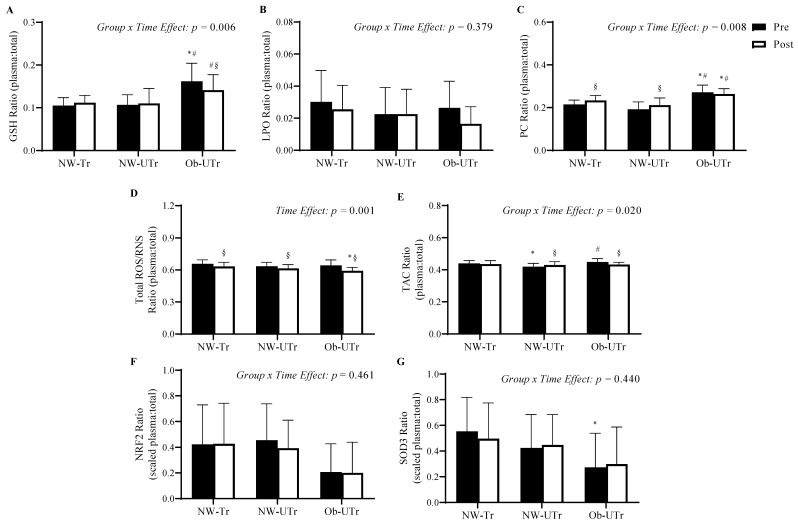
Proportion of plasma relative to total circulating oxidative stress concentrations (plasma plus ELV) prior to and in response to acute maximal treadmill exercise. Repeated measures analysis of variance analysis demonstrated a differential impact of acute maximal treadmill exercise on the ratios of GSH (panel **A**), PC (panel **C**), and TAC (panel **E**) in Ob-UTr compared to NW-Tr and NW-Utr participants, whereas the ratio of total ROS/RNS was similar between participant groups (panel **D**). Acute maximal treadmill exercise exhibited no impact on the ratio LPO (panel **B**), NRF2 (panel **F**), or SOD3 (panel **G**) across participant groups. Data are presented as means ± S.D. * indicates a significant difference compared to NW-Tr participants; ^#^ indicates a significant difference compared to NW-Utr participants; ^§^ indicates a significant difference compared to pre-exercise values *(p* ≤ 0.05). Abbreviations: ELV: exosome-like extracellular vesicles; GSH: glutathione; LPO: lipid peroxidation; NRF2: nuclear factor erythroid 2-related factor 2; NW-Tr: individuals with normal weight who are aerobically trained; NW-UTr: individuals with normal weight who are aerobically untrained; Ob-UTr: individuals with obesity who are aerobically untrained; PC: protein carbonyl; ROS/RNS: reactive oxygen/nitrogen species; SOD3: superoxide dismutase 3; TAC: total antioxidant capacity.

Next, the ratios of plasma-to-total (plasma plus ELV) oxidative stress concentrations were examined prior to and in response to acute maximal treadmill exercise. Prior to maximal exercise, the ratios of plasma-to-total GSH and PC concentrations were significantly greater in Ob-UTr compared to both NW-Tr and NW-Utr participants (*F*_[2, 39]_ = 15.645, *p* < 0.001, η^2^ = 0.45; *F*_[2, 39]_ = 22.022, *p* < 0.001, η^2^ = 0.53, respectively; Figure 4A,C). In addition, TAC values were lower in NW-Tr and greater in Ob-Utr relative to NW-Utr participants (*F*_[2, 39]_ = 8.203, *p* < 0.001, η^2^ = 0.30; Figure 4E), and SOD3 were greater in NW-Tr compared to Ob-Utr participants (*F*_[2, 39]_ = 3.448, *p* = 0.042, η^2^ = 0.15; Figure 4G). No differences were observed in baseline ratios of plasma-to-total LPO (*F*_[2, 39]_ = 0.737, *p* = 0.485, η^2^ = 0.04; Figure 4B), total ROS/RNS concentrations (*F*_[2, 39]_ = 1.178, *p* = 0.319, η^2^ = 0.06; Figure 4D), or NRF2 (*F*_[2, 39]_ = 2.724, *p* = 0.078, η^2^ = 0.12; Figure 4F). 

In response to acute maximal treadmill exercise, significant decreases in the ratio of plasma-to-total GSH concentrations were observed in Ob-UTr compared to NW-Tr and NW-UTr participants (*Group* × *Time Effect*: *F*_[2, 39]_ = 5.875, *p* = 0.006, η^2^ = 0.23), whereas significant increases in the ratio of plasma-to-total PC concentrations were observed in NW-Tr and NW-UTr compared to Ob-UTr participants (*Group* × *Time Effect*: *F*_[2, 39]_ = 5.404, *p* = 0.008, η^2^ = 0.22). In addition, although the ratio of plasma-to-total ROS/RNS concentrations decreased similarly across all participant groups (*Time Effect: F*_[1, 39]_ = 15.993, *p* < 0.001, η^2^ = 0.29), the ratio of plasma-to-total TAC values increased in NW-UTr and decreased in Ob-UTr participants only (*Group* × *Time Effect*: *F*_[2, 39]_ = 4.332, *p* = 0.020, η^2^ = 0.18). No differences in the ratio of plasma-to-total LPO (*Group × Time Effect: F*_[2, 39]_ = 0.994, *p* = 0.379, η^2^ = 0.05), NRF2 (*Group* × *Time Effect*: *F*_[2, 39]_ = 0.789, *p* = 0.461, η^2^ = 0.04), or SOD3 (*Group* × *Time Effect*: *F*_[2, 39]_ = 0.839, *p* = 0.440, η^2^ = 0.04) were observed.

### 3.6. Associations among BMI and Cardiorespiratory Fitness with Indices of Oxidative Stress

**Figure 5:**
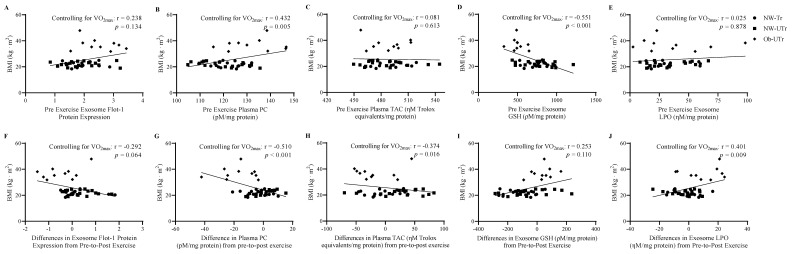
Associations among BMI with ELV markers and oxidative stress measured in plasma and from circulating ELVs. To prevent the potential impact of cardiorespiratory fitness levels on these relationships, correlational analyses were examined while controlling for relative VO_2max_. ELV markers: ELV Flot-1 expression was not associated with BMI prior to or in response to acute maximal treadmill exercise (panels **A** and **F**). Oxidative stress in plasma: PC concentrations were positively associated with BMI prior to exercise and negatively associated with BMI in response to acute maximal treadmill exercise (panels **B** and **G**). Although no associations were observed between BMI and TAC values prior to exercise, a negative association was observed in response to acute maximal treadmill exercise (panels **C** and **H**). Oxidative stress in circulating ELVs: ELV GSH concentrations were negatively associated with BMI prior to exercise, but not in response to acute maximal treadmill exercise (panels **D** and **I**). Although no associations were observed between BMI and ELV LPO concentrations prior to exercise, a positive association was observed in response to acute maximal treadmill exercise (panels **E** and **J**). Abbreviations: BMI: body mass index; ELV: exosome-like extracellular vesicles; Flot-1: flotillin-1; GSH: glutathione; LPO: lipid peroxidation; PC: protein carbonyl; TAC: total antioxidant capacity; VO_2max_: cardiorespiratory fitness.

Next, the relationships of BMI and relative VO_2max_ with absolute changes (from pre-to-post acute maximal exercise) in ELV markers and indices of oxidative stress were examined across all participant groups. Analyses were focused on the changes in ELV Flot-1 expression, plasma PC concentrations, and TAC values, and circulating ELV GSH and LPO concentrations as these variables responded differently across participant groups in response to maximal exercise. At baseline, no association was observed between ELV Flot-1 expression and BMI (r = 0.238, *p* = 0.134; Figure 5A). On the contrary, BMI was positively associated with plasma PC concentrations (r = 0.432, *p* = 0.005; Figure 5B), but not TAC values (r = 0.081, *p* = 0.613; Figure 5C) and negatively associated with ELV GSH expression (r = −0.551, *p <* 0.001; Figure 5D), but not ELV LPO concentrations at baseline (r = 0.025, *p* = 0.878; Figure 5E). In response to maximal exercise, BMI tended to be negatively associated with ELV Flot-1 expression (r = −0.292, *p* = 0.064; Figure 5F). In addition, BMI was negatively associated with changes in plasma PC concentrations and TAC values (r = −0.510, *p* < 0.001; r = −0.374, *p* = 0.016, respectively; Figure 5G,H). Although BMI was not associated with changes in ELV GSH concentrations (r = 0.253, *p* = 0.110; Figure 5I), a positive association was observed for changes in ELV LPO concentrations (r = 0.041, *p =* 0.009; Figure 5J). Finally, no associations between relative VO_2max_ were observed with Flot-1 at baseline or in response to maximal exercise (r = −0.061, *p* = 0.706; r = −0.009, *p* = 0.958, respectively). Similarly, no associations at baseline or in response to maximal exercise were observed between relative VO_2max_ or plasma PC concentrations (r = 0.187, *p* = 0.243; r = −0.181, *p* = 0.257, respectively), TAC values (r = 0.157, *p* = 0.326; r = −0.306, *p* = 0.052, respectively), or ELV GSH (r = −0.147, *p* = 0.358; r = −0.198, *p* = 0.214, respectively) and LPO concentrations (r = −0.105, *p* = 0.513; r = 0.117, *p* = 0.464, respectively).

**Figure 6:**
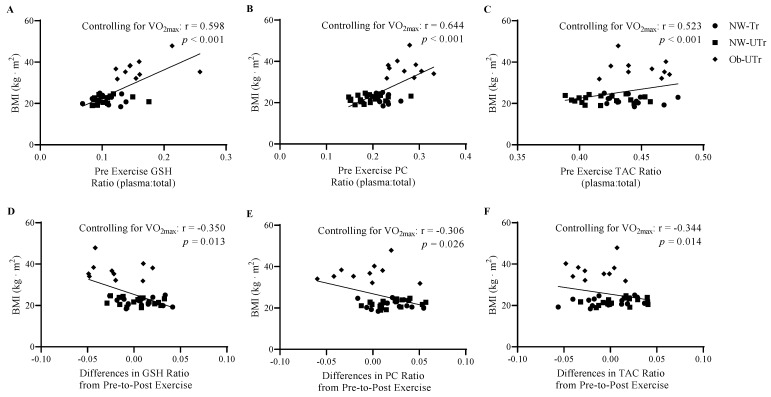
Associations among BMI with proportion of plasma-to-total circulating oxidative stress concentrations (plasma plus ELVs) were measured prior to and in response to acute maximal treadmill exercise (expressed as absolute change relative to pre-exercise values). To prevent the potential impact of cardiorespiratory fitness levels on these relationships, correlational analyses were examined while controlling for relative VO_2max_. BMI was positively associated with the ratio of GSH, PC, and TAC prior to exercise (panels **A**–**C**) and negatively associated with GSH, PC, and TAC ratio changes in response to acute maximal treadmill exercise (panels **D**–**F**). Abbreviations: BMI: body mass index; GSH: glutathione; LPO: lipid peroxidation; PC: protein carbonyl; VO_2max_: cardiorespiratory fitness.

Lastly, the relationships of BMI and relative VO_2max_ with the ratios of plasma-to-total GSH and PC concentrations and TAC values were examined prior to acute maximal treadmill exercise. In addition, the change in ratios in response to exercise was examined. At baseline, BMI (controlling for VO_2max_) was positively associated with the ratios of plasma-to-total GSH (r = 0.598, *p* < 0.001; Figure 6A), PC (r = 0.644, *p* < 0.001; Figure 6B), and TAC (r = 0.523, *p* < 0.001; Figure 6C). In response to acute maximal treadmill exercise, BMI was negatively associated with changes in the ratios of plasma-to-total GSH (r = −0.350, *p* = 0.013; Figure 6D), PC (r = −0.306, *p* = 0.026; Figure 6E), and TAC (r = −0.344, *p* = 0.014; Figure 6F). Although VO_2max_ (controlling for BMI) was not associated with the ratios of plasma-to-total GSH (r = 0.046, *p* = 0.389), a positive association was observed with PC (r = 0.269, *p =* 0.045) and TAC (r = 0.476, *p* = 0.001). In response to acute maximal treadmill exercise, no associations were observed between VO_2max_ and changes in the ratios of plasma-to-total GSH (r = 0.089, *p* = 0.291) PC (r = −0.023, *p* = 0.444), or TAC (r = −0.258, *p* = 0.052). 

## 4. Discussion

This study investigated the impact of cardiorespiratory fitness and obesity on ELV markers and oxidative stress. The main findings demonstrate that obesity (BMI) is associated with elevated concentrations of plasma PC concentrations at baseline and a significantly greater reduction in plasma PC concentrations and TAC values in response to acute maximal treadmill running. Additionally, BMI is associated with reduced ELV GSH concentrations and, in response to exercise, greater increases in ELV LPO concentrations. Finally, the ratios of plasma-to-total (plasma plus ELV) GSH, PC, and TAC are positively associated with BMI prior to exercise and negatively associated with BMI in response to acute maximal treadmill exercise. These findings suggest that BMI, independently of cardiorespiratory fitness levels, impacts ELV marker expression and oxidative stress following acute exercise.

The “exercise secretome”, exosomes included, represents a biochemical signaling mechanism by which various organs and cell/tissue activated during physical activity and exercise communicate with other organ and cell/tissues throughout the body [14]. Previous studies have demonstrated an increase in exosome release into circulation following acute exercise [19]. However, a recent review by Estébanez et al. suggests that results from human studies have produced mixed results, potentially due to the differences in exercise protocols, study populations, or methods to isolate and examine exosomes utilized across the literature [10]. Nonetheless, the present study demonstrates that Flot-1 expression levels from circulating plasma ELVs were greater in Ob-UTr compared to both the NW-Tr and NW-Utr groups at baseline. Hou et al. have also previously indicated that plasma exosome concentrations were not different among NW-Tr compared to NW-Utr males [22]. More recently, both 8 weeks of resistance training in healthy elderly participants and 12 weeks of submaximal aerobic training (60–70% heart rate reserved) combined with resistance exercise 3 days per week among healthy obese participants have been shown to exhibit no impact of Flot-1 exosome expression [29]. These findings suggest that BMI, but not cardiorespiratory fitness or aerobic training status, might serve as a larger regulator of plasma exosomes while at rest. 

In response to acute maximal exercise, only NW-UTr participants exhibited a significant increase in ELV Flot-1 expression relative to the resting levels. Previous studies have also demonstrated that maximal treadmill exercise elicits an immediate and sustained Flot-1 increase in four moderately trained males [20]. However, the extremely small sample size and lack of comparative populations are limiting, and comparisons across these studies should be considered accordingly. Nonetheless, BMI tended to be negatively associated with changes in ELV Flot-1 expression in the present study, which, in part, is supported by recent evidence that plasma exosome release is greater in NW-UTr compared to Ob-UTr participants following an acute bout of submaximal treadmill running (60% VO_2max_) [25]. It is important to note that the exosome response has been shown to be transient, and the present study only examined ELVs prior to and immediately following acute exercise. Thus, it is unknown if ELV kinetics would differ between participant groups throughout recovery from acute exercise. Likewise, interest in plasma ELV concentrations and marker expression might be too narrowly focused. More specifically, Hou et al. demonstrate that despite similar exosome concentrations, the cardioprotective function of exosomes isolated from the aerobically trained participants was augmented compared to the aerobically untrained group while at rest [22]. Although the impact of acute exercise on exosomes was not examined in by Hou and colleagues, these results suggest that cargo being transported under resting conditions reflects the health benefits of regular physical activity and exercise training. Therefore, future studies should examine differences in the ex vivo functional capacity of isolated exosomes in obese populations to determine whether the increased expression observed at baseline is compensating for altered functionality and, second, if the potential health-mediating benefits can be augmented following aerobic exercise (and dietary) interventions, independent of exosome marker expression patterns and changes in BMI. 

Another important finding from the present study is the impact of BMI on oxidative stress in plasma and from circulating ELVs. For example, it is well established that obesity is associated with altered indices of oxidative stress at rest and in response to aerobic exercise [11]. Interestingly, only plasma PC concentrations were elevated in participants with obesity, whereas no other differences prior to exercise were observed. Although unexpected, this could point to the otherwise young and healthy nature of the populations under investigation. Nonetheless, BMI in the present study was associated with elevated plasma PC concentrations at baseline and a significantly greater reduction in plasma PC concentrations and TAC values in response to acute maximal treadmill running. Interestingly, the relationship between BMI and indices of oxidative stress in circulating exosomes revealed an opposite pattern of association compared to plasma concentrations. More specifically, ELV GSH and PC concentrations were lower in Ob-UTr compared to both NW-Tr and NW-UTr groups at baseline, and changes in LPO concentrations were elevated in the Ob-UTr groups following acute maximal treadmill exercise. 

The release of ELVs into circulation following physical stress represents an important mechanism in response to oxidative stress by delivering readily available antioxidants and substrate for LPO [23]. LPO represents an important marker of obesity-related inflammation and disease potential [4], and the increased concentration of LPO within exosomes has been shown to act as an endogenous activator of the Toll-like receptor 4 pathway to elicit an elevated pro-inflammatory response via the nuclear factor κB transcription factor [24]. As a result, the increased plasma ELV LPO concentrations in individuals with obesity may serve as a danger-associated molecular pattern that increases activation of the innate immune response following acute exercise, thereby supporting the notion that obesity is a chronic, low-grade inflammatory condition associated with increased risk for disease pathology. Given the known impact of physical activity and aerobic exercise on eliciting an anti-inflammatory phenotype and improving antioxidant capacity under resting conditions and in response to acute aerobic exercise [7,30], it is also possible that routine engagement in physical activity or voluntary aerobic exercise training might help mitigate against the observed oxidative stress response in young adults with obesity. 

Less understood are the lower ELV GSH concentrations. For example, increased indices of inflammation have been linked to lower GSH concentrations in human plasma and immune cells as well as reduced expression in rodent skeletal muscle [31,32]. As a result, the chronic low-grade inflammatory state associated with obesity could negatively impact the effectiveness of weight management and weight loss strategies in individuals with obesity [33]. Likewise, the acute nature of this study prevents a more accurate interpretation of these results, which, combined with lower ELV PC concentrations, highlights the complexity of these results and the dynamics of the oxidative stress response in general. Furthermore, these results are limited by the overall small sample size and more so by the lack of aerobically trained participants with obesity in the study. Without such a comparative control population with obesity, it is unknown whether the role dysregulation of LPO, GSH, and PC observed in the present study is a result of having obesity or a combination of having obesity in the absence of sufficient cardiorespiratory fitness. Thus, future studies should aim to include an aerobically trained population with obesity to address this limitation. Furthermore, an understanding of how the release of cargo, such as indices of oxidative stress, from exercise-mediated exosomes is altered with increased cardiorespiratory fitness or exercise training would help determine the mechanisms by which exosomes regulate the pathology of obesity-related diseases under resting or exercised conditions [34].

Further supporting the impact of obesity on indices of oxidative stress in plasma and circulating ELVs, the ratios of plasma-to-total (plasma plus ELV) GSH and PC concentrations and TAC values were greater in Ob-UTr compared to NW-Tr (GSH and PC only) and NW-UTr participants (all three indices). Similarly, in contrast to the positive associations observed with BMI, greater decreases in the ratios of plasma-to-total GSH concentrations and TAC values, as well as a blunted increase in PC plasma-to-total ratios, were observed in Ob-UTr relative to NW-Tr and NW-UTr participants. These findings suggest that the capacity to regulate an antioxidant defense, as observed in plasma, is altered in the presence of obesity. Alternatively, differences in the observed ratios of plasma-to-total oxidate stress concentrations between Ob-UTr relative to both NW-Tr and NW-UTr participant groups potentially indicate that acute maximal treadmill exercise mobilizes a more robust antioxidant defense via circulating ELVs. This slight shift from plasma to ELV concentrations may represent a mechanism by which skeletal muscle therapeutically benefits nearby target tissue in response to physical activity and exercise to compensate for obesity-related health consequences. 

In conclusion, results from this study demonstrate that BMI, independent of VO_2max_, differentially regulates ELV Flot-1 expression and indices of oxidative stress within plasma and circulating ELVs prior to and in response to a single session of maximal treadmill exercise. On the contrary, increased relative VO_2max_ does not appear to significantly impact ELV marker expression or indices of oxidative stress relative to untrained participants with lower cardiorespiratory fitness levels. Future studies should consider the physiological impact of isolated ELVs, independent of relative concentrations measured in circulation, to determine their potential impact on cell-to-cell communication at rest and whether these responses are altered in response to acute and chronic aerobic exercise. Such investigation will better serve to inform the ongoing efforts to determine the biological significance of exosomes and the overall “exercise secretome”, and moreover, the differential impact of obesity and cardiorespiratory fitness levels on the potential health-mediating benefits they exhibit in response to acute and chronic aerobic exercise.

## Figures and Tables

**Table 1:** Participant descriptive characteristics.

Variable	NW-Tr (n = 15)	NW-Utr (n = 18)	Ob-UTr (n = 10)	*p*-Value
Age	25.27 ± 4.57	23.02 ± 3.25	26.76 ± 5.05	0.073
Weight (kg)	69.42 ± 6.35	70.53 ± 9.23	115.62 ± 15.98 *^#^	<0.001
Height (m)	1.79 ± 0.05	1.78 ± 0.08	1.77 ± 0.07	0.708
BMI (kg/m^2^)	21.68 ± 1.98	22.16 ± 1.83	36.98 ± 4.69 *^#^	<0.001
Waist (cm)	75.49 ± 3.91	78.58 ± 6.35	111.55 ± 13.17 *^#^	<0.001
Hip (cm)	95.41 ± 3.46	96.44 ± 5.84	118.23 ± 7.29 *^#^	<0.001
Waist-to-Hip Ratio	0.791 ± 0.027	0.815 ± 0.048	0.940 ± 0.081 *^#^	<0.001
Resting HR (bpm)	60.2 ± 5.35	69.5 ± 9.39 *	75.60 ± 6.50 *^#^	<0.001
Resting SBP (mmHg)	117.33 ± 8.27	111.78 ± 7.76	134.20 ± 8.24 *^#^	<0.001
Resting DBP (mmHg)	78.40 ± 9.89	74.44 ± 9.09	84.40 ± 6.38 ^#^	0.025
Absolute VO_2_ (L·min^−1^)	4.37 ± 0.41	3.14 ± 0.47 *	4.12 ± 0.72 ^#^	<0.001
Relative VO_2_ (mL·kg^−1^·min^−1^)	63.03 ± 4.29	44.61 ± 3.86 *	36.34 ± 6.13 *^#^	<0.001

**Note:** Data are presented as means ± S.D. * indicates a significant difference compared to NW-Tr participants; # indicates a significant difference compared to NW-UTr participants. Significance was determined with one-way ANOVA (*p* ≤ 0.05). Abbreviations: BMI: body mass index; DBP: diastolic blood pressure; HR: heart rate; NW-Tr: individuals with normal weight who are aerobically trained; NW-UTr: individuals with normal weight who are aerobically untrained; Ob-UTr: individuals with obesity who are aerobically untrained; SBP: systolic blood pressure; VO_2_: maximal oxygen consumption.

## Data Availability

Data are available upon request.

## Supplementary Materials

The following supporting information can be downloaded at: https://www.mdpi.com/article/10.3390/biology13080599/s1, Figure S1: The uncropped western blot figures were presented in Figure S1; Table S1: Western Blot Ratios normalized to Ponceau S.
