# Supplementary material for: Impact of BMI and Cardiorespiratory Fitness on Oxidative Stress in Plasma and Circulating Exosomes Following Acute Exercise
Source: Biology (Basel). 2024 Aug 8;13(8):599. doi: 10.3390/biology13080599 (PMC11352065; doi:10.3390/biology13080599)
Supplement: Supplementary file 1 [file biology-13-00599-s001.zip › Figure S1.pptx]

## Slide 1
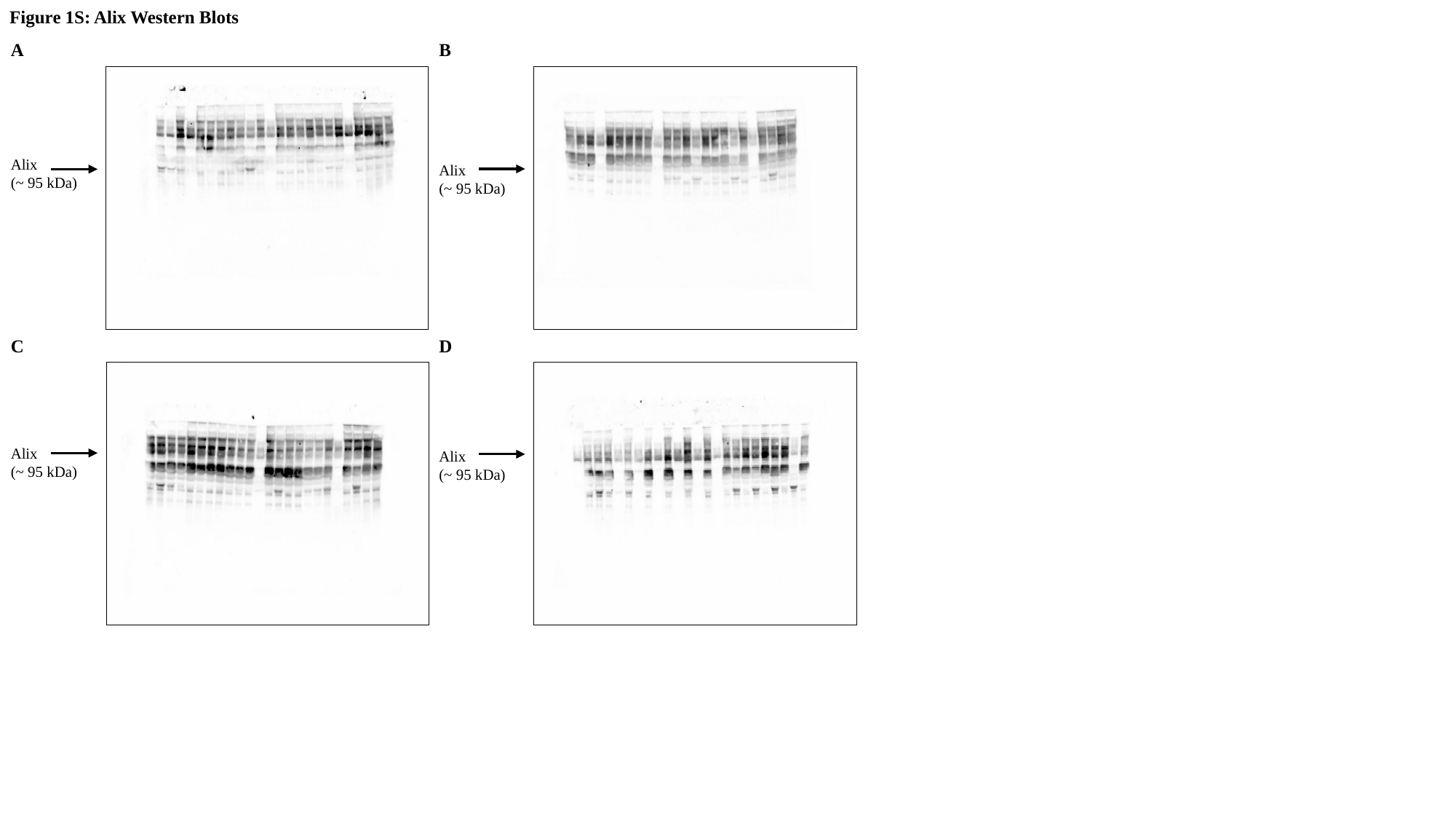

Figure 1S: Alix Western Blots
B
A
Alix
(~ 95 kDa)
Alix
(~ 95 kDa)
C
D
Alix
(~ 95 kDa)
Alix
(~ 95 kDa)

## Slide 2
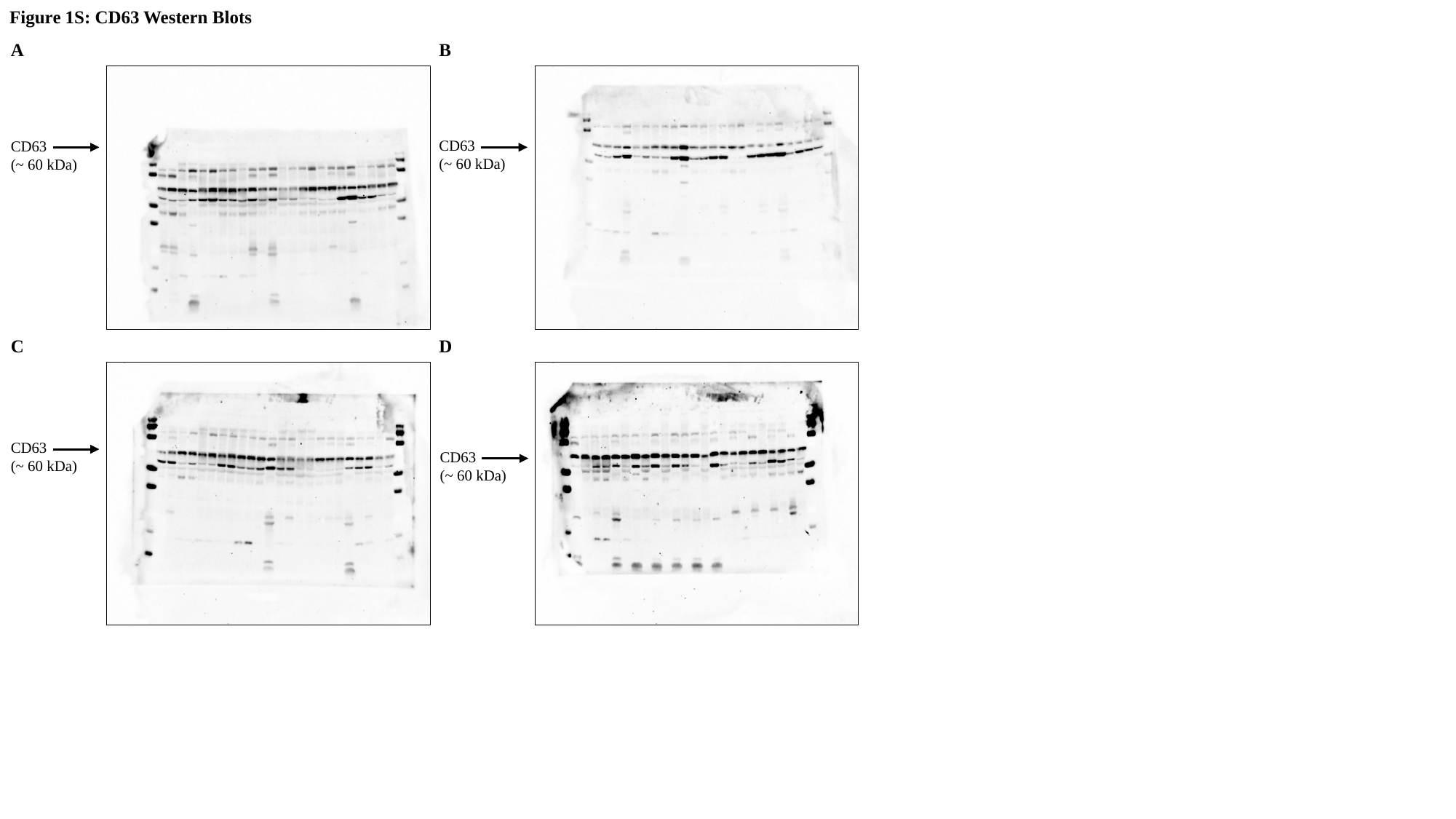

Figure 1S: CD63 Western Blots
B
A
CD63
(~ 60 kDa)
CD63
(~ 60 kDa)
C
D
CD63
(~ 60 kDa)
CD63
(~ 60 kDa)

## Slide 3
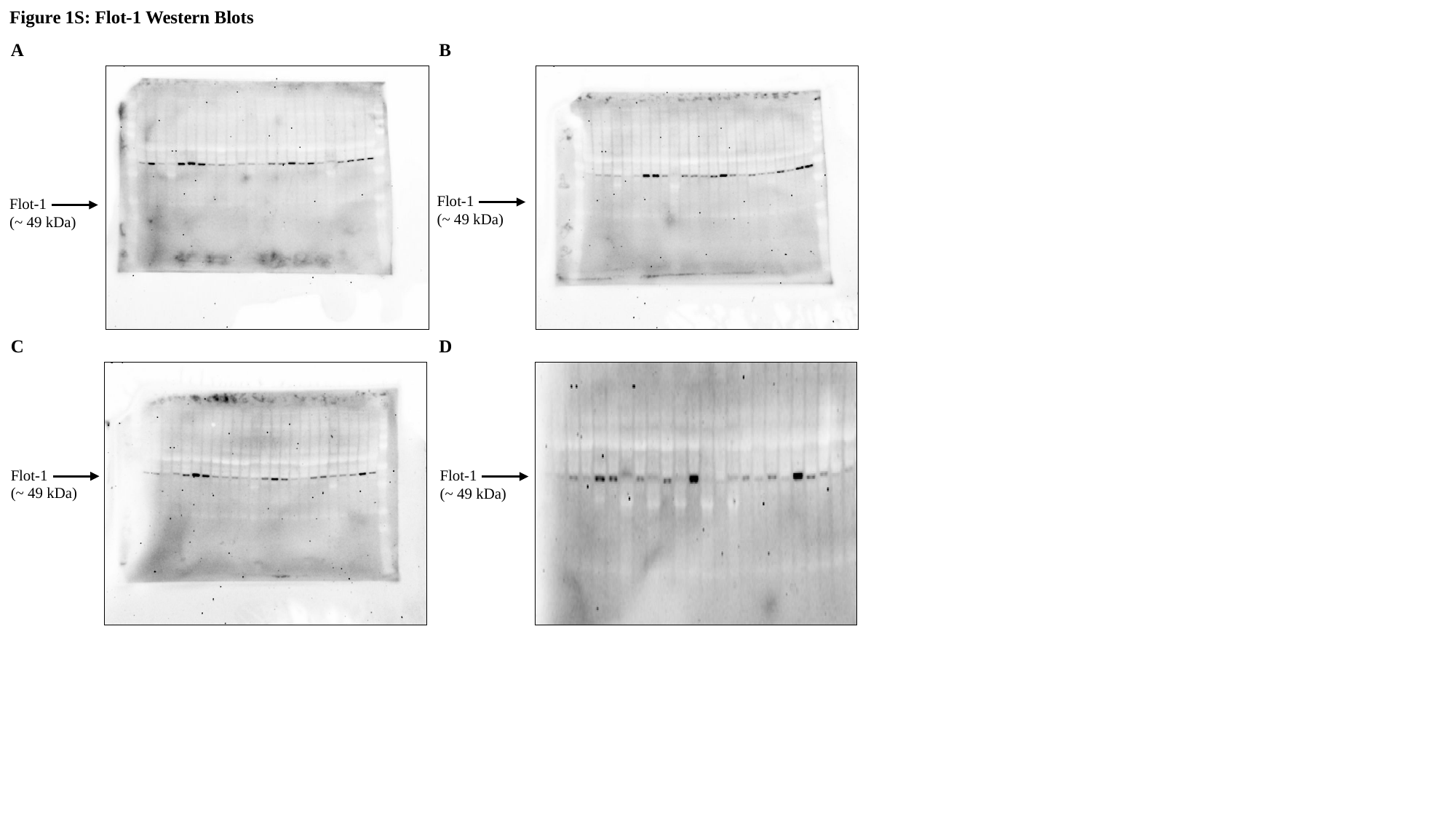

Figure 1S: Flot-1 Western Blots
B
A
Flot-1
(~ 49 kDa)
Flot-1
(~ 49 kDa)
C
D
Flot-1
(~ 49 kDa)
Flot-1
(~ 49 kDa)

## Slide 4
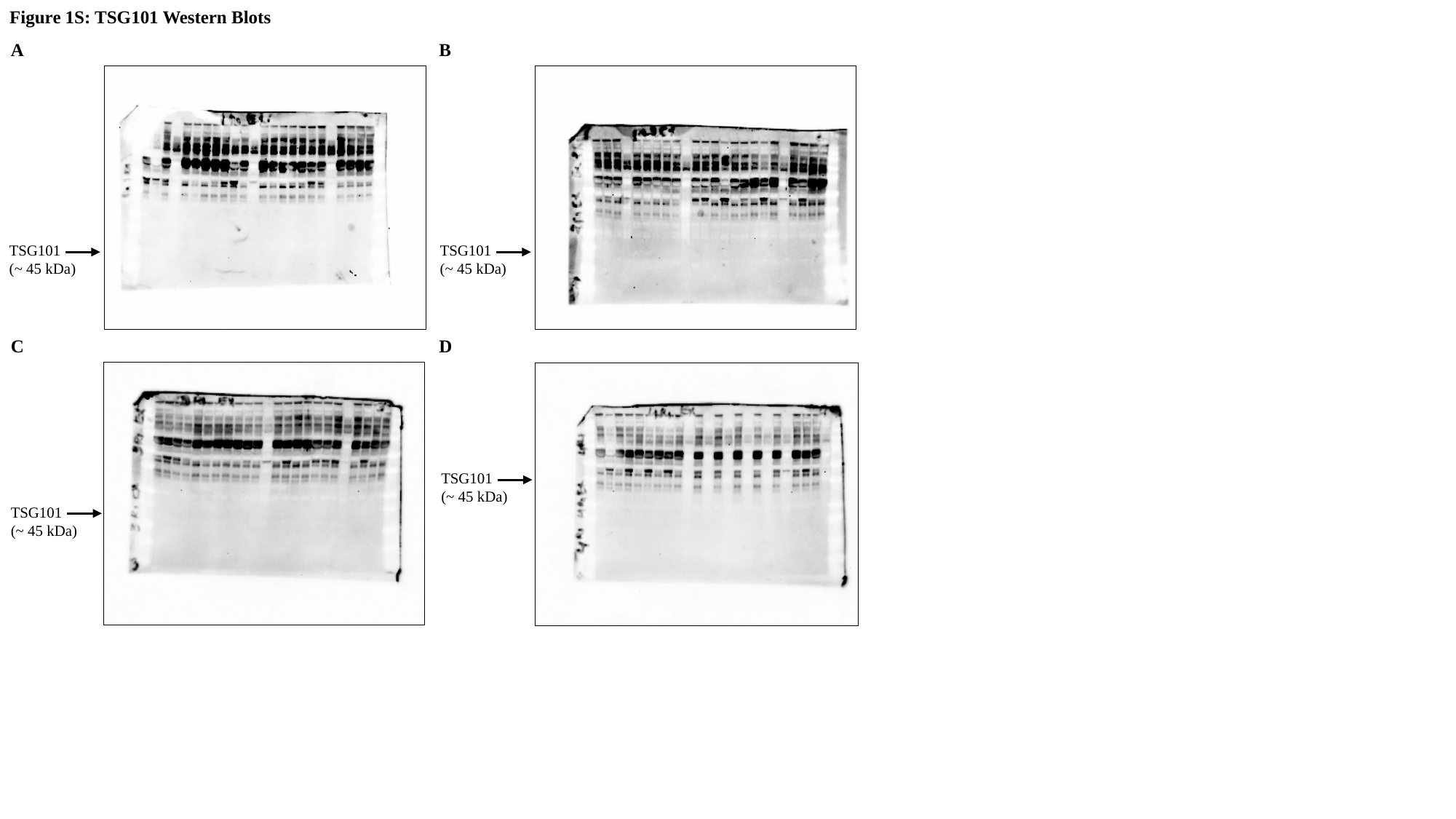

Figure 1S: TSG101 Western Blots
B
A
TSG101
(~ 45 kDa)
TSG101
(~ 45 kDa)
C
D
TSG101
(~ 45 kDa)
TSG101
(~ 45 kDa)

## Slide 5
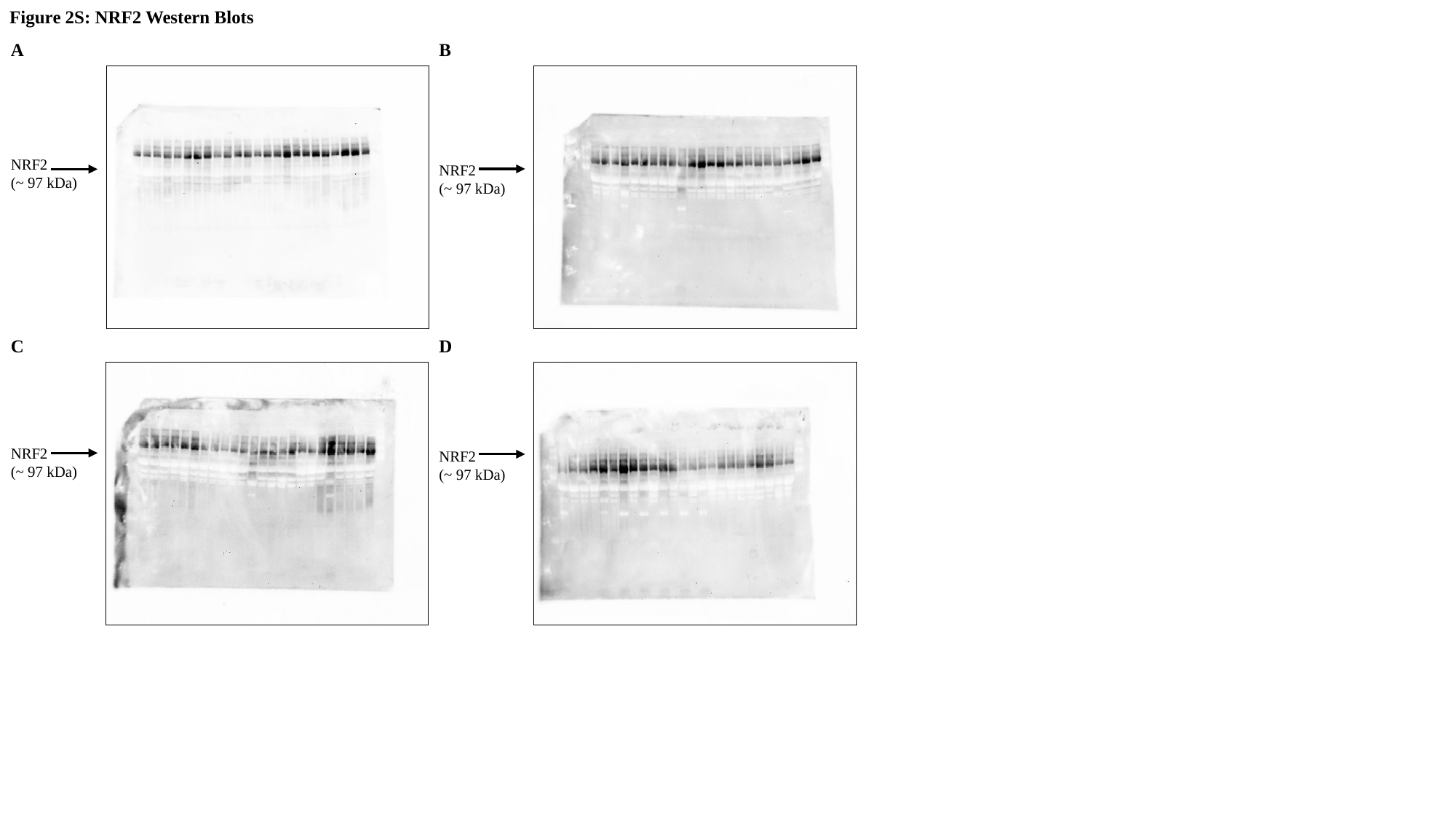

Figure 2S: NRF2 Western Blots
B
A
NRF2
(~ 97 kDa)
NRF2
(~ 97 kDa)
C
D
NRF2
(~ 97 kDa)
NRF2
(~ 97 kDa)

## Slide 6
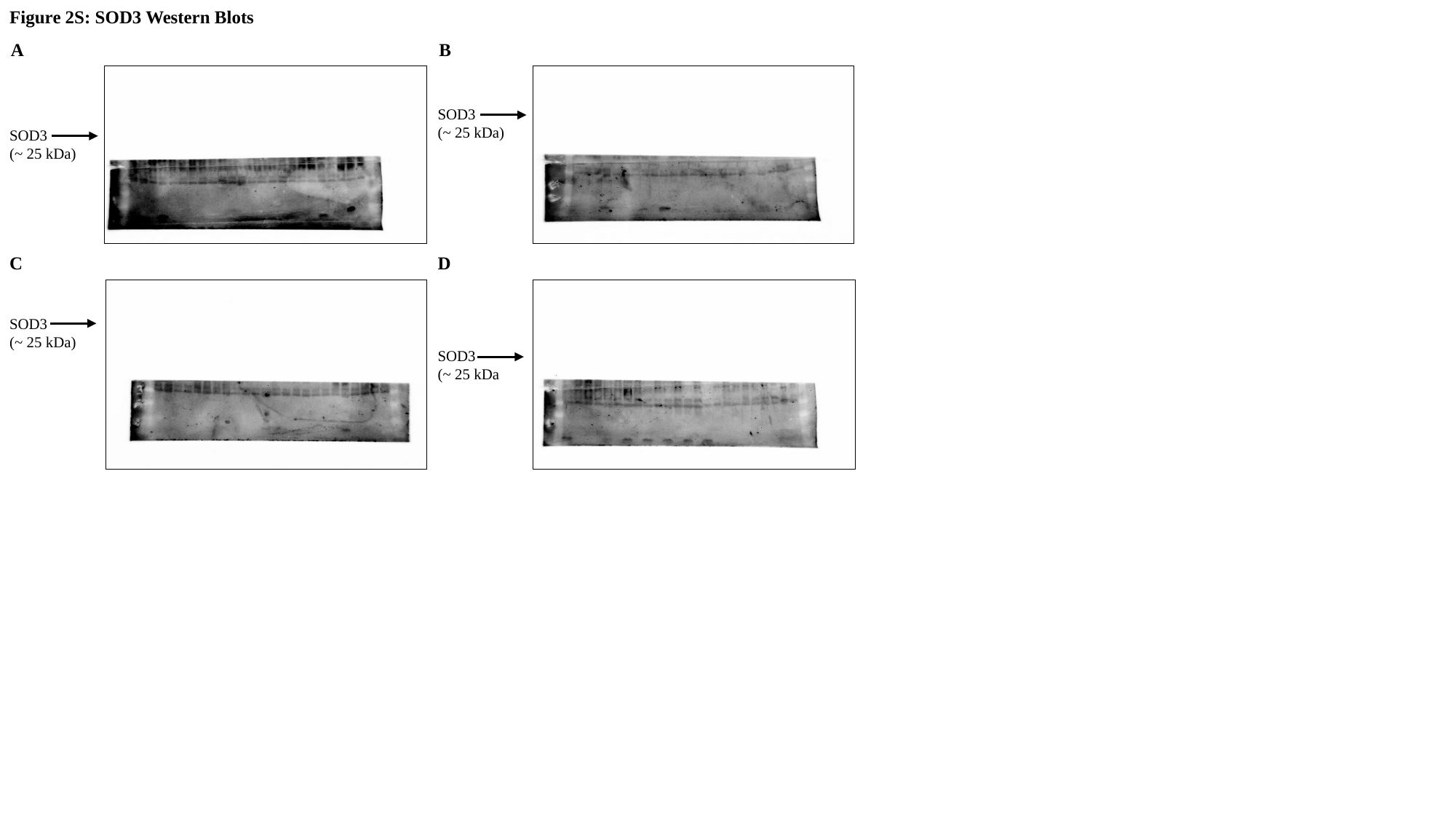

Figure 2S: SOD3 Western Blots
B
A
SOD3
(~ 25 kDa)
SOD3
(~ 25 kDa)
C
D
SOD3
(~ 25 kDa)
SOD3
(~ 25 kDa

## Slide 7
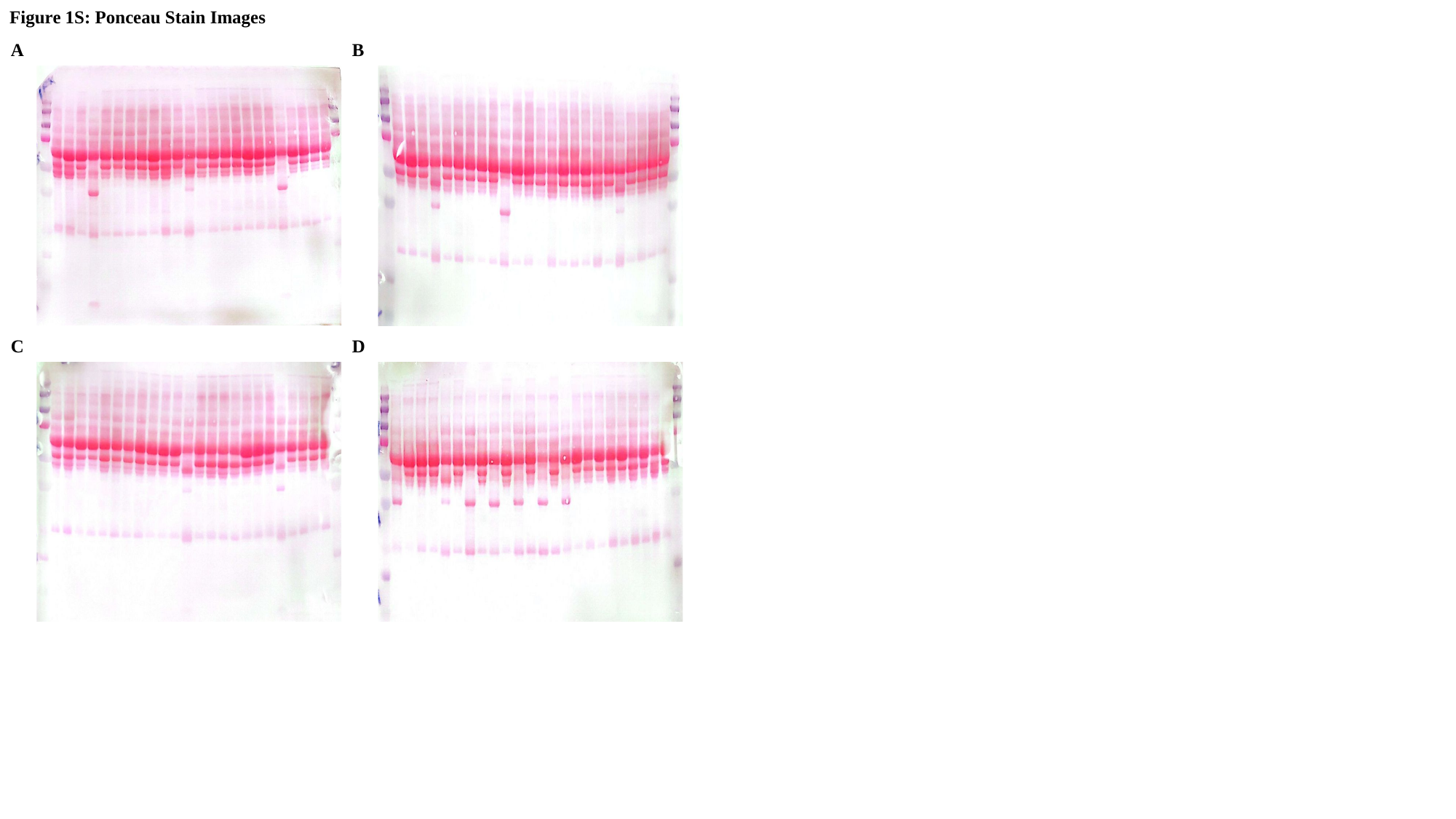

Figure 1S: Ponceau Stain Images
B
A
D
C
